# Cost-effectiveness of zinc supplementation for prevention of childhood diarrhoea in Tanzania

**DOI:** 10.1017/S1368980022000568

**Published:** 2022-07

**Authors:** Happiness Pius Saronga, Karim Manji, Enju Liu, Christopher P Duggan, Nicolas A Menzies

**Affiliations:** 1 Behavioural Sciences Department, School of Public Health and Social Sciences, Muhimbili University of Health and Allied Sciences, 65001 Dar-es-salaam, Tanzania; 2 Department of Paediatrics and Child Health, School of Medicine, Muhimbili University of Health and Allied Sciences, Dar-es-salaam, Tanzania; 3 Division of Gastroenterology, Hepatology, and Nutrition, Boston Children’s Hospital, Harvard Medical School, Boston, MA, USA; 4 Institutional Centers for Clinical and Translational Research, Boston Children’s Hospital, Boston, MA, USA; 5 Department of Nutrition, Harvard T.H Chan School of Public Health, Boston, MA, USA; 6 Department of Global Health and Population, Harvard T.H Chan School of Public Health, Boston, MA, USA

**Keywords:** Diarrhoea, Zinc, Supplementation, Children, Cost-effectiveness, Tanzania

## Abstract

**Objective:**

To assess the cost-effectiveness of prophylactic Zn supplementation for preventing diarrhoea in young children in Tanzania.

**Design:**

Cost-effectiveness analysis using decision-analytic modelling. Cost-effectiveness ratios were calculated as the incremental cost (2019 USD) per disability-adjusted life year (DALY) averted, from a societal perspective, and with a 3 % discount rate applied to future outcomes. Sensitivity analyses were performed to test the robustness of results to alternative assumptions.

**Setting:**

Tanzania.

**Participants:**

A hypothetical cohort of 10 000 children aged 6 weeks to 18 months.

**Results:**

The intervention costs of Zn supplementation were estimated as $109 800 (95 % uncertainty interval: 61 716, 171 507). Zn supplementation was estimated to avert 2200 (776, 3737) diarrhoeal episodes, 14 080 (4692, 25 839) sick days, 1584 (522, 2927) outpatient visits, 561 (160–1189) inpatient bed days, 0·51 (0·15, 1·03) deaths and 19·3 (6·1, 37·5) DALY (discounted at 3 % per year). Zn supplementation reduced diarrhoea care costs by $12, 887 (4089, 25 058). The incremental cost per DALY averted was $4950 (1678, 17 933). Incremental cost-effectiveness ratios (ICER) estimated from a health system perspective were similar to the results from the societal perspective. ICER were substantially lower (more favourable) when future outcomes were not discounted, but all ICER were above contemporary thresholds for cost-effectiveness in this setting.

**Conclusion:**

Prophylactic Zn reduced diarrhoea incidence and associated healthcare utilisation; however, it did not appear to be cost-effective for prevention of childhood diarrhoea in the scenario examined in this study. Reducing intervention costs, or identifying high risk groups for intervention targeting, may be needed to improve cost-effectiveness in this setting.

Diarrhoea is among the top three causes of death and illness in children under 5 years of age in Tanzania^(1)^. In the 2015–2016 Demographic and Health Survey, 12 % of children in this age group were reported to have had diarrhoea in the 2 weeks before the survey^(2)^. Malnutrition has been linked to diarrhoea in children, with children suffering from malnutrition at increased risk of diarrhoea due to compromised immunity^(3)^. Diarrhoea can also exacerbate malnutrition, leading to on-going co-morbid diarrhoea and malnutrition. Zn deficiency is common in low-income countries due to poor dietary intake and limited bioavailability and is associated with increased risk of gastrointestinal infections causing diarrhoea^(4,5)^. Zn is a vital micronutrient important for protein synthesis, cell growth and differentiation, immune function and intestinal transport of water and electrolytes. It is associated with many other positive health benefits necessary for growth and development^(6,7)^.

Zn supplements can be given to children therapeutically or prophylactically^(5)^. Zn and oral rehydration solution are the standard for treatment of childhood diarrhoea, as recommended by the WHO^(8)^. Several studies have also shown the effectiveness of Zn given to healthy children as a diarrhoea prophylaxis^(5–7)^. Despite this evidence, Zn is not currently recommended for childhood diarrhoea prophylactic purposes. Prophylactic Zn could potentially be used alongside rotavirus vaccine to avert childhood diarrhoea, especially among the neediest children with inadequate diet, for whom Zn supplementation might be most beneficial.

In Tanzania, a randomised, double-blind, placebo-controlled clinical trial (NCT00421668) found that daily Zn supplementation was effective in preventing diarrhoea among young children aged 6 weeks to approximately 18 months^(9)^. In this trial, children were randomly assigned to receive daily Zn supplementation or placebo for 18 months and followed up monthly to determine the effect of Zn on diarrhoea incidence. Prophylactic Zn supplementation was found to reduce the occurrence of all types of diarrhoea (12 % reduction, rate ratio 0·88 (0·81, 0·96)) and dysentery (16 % reduction, rate ratio 0·84 (0·74, 0·95)).

There is limited information with regard to the cost-effectiveness of prophylactic Zn supplementation. While a number of studies have examined the cost-effectiveness for preventing diarrhoea^(10–12)^, these studies have been performed in the context of therapeutic Zn supplementation, and the outcomes for prophylactic Zn may differ. In addition, the few cost-effectiveness studies for prophylactic Zn have been conducted among children older than 6 months of age^(13–15)^ and none has been performed using data from East Africa. This study was therefore conducted to fill the knowledge gap with regard to the cost-effectiveness of prophylactic Zn supplementation in preventing various forms of diarrhoea in children aged between 6 weeks and 18 months in an East African setting. A decision-analytic model of Zn supplementation for young children in Tanzania was tested to determine the cost-effectiveness of prophylactic Zn supplementation.

## Methods

### Analytic approach

We investigated the incremental costs and benefits of preventive Zn supplementation using a model parameterised with local Tanzanian data on costs, diarrhoeal disease epidemiology and effectiveness of Zn supplementation. The model was used to estimate costs and health outcomes for a hypothetical cohort of 10 000 children aged 6 weeks to 18 months under different scenarios. The incremental cost-effectiveness of preventive Zn supplementation was calculated as the difference in costs and health outcomes between a scenario with preventive Zn supplementation as compared with a scenario describing current standard-of-care (no supplementation).

### Hypothetical Zn supplementation programme

For the Zn supplementation programme, we assumed that children up to 6 months of age would receive one 5 mg Zn capsule per day (250 % of the Adequate Intake in this age group)^(9)^, and children 7–18 months of age would receive two 5 mg capsules per day (333 % of the RDA)^(16)^. It was assumed Zn supplementation would be delivered with the same approach as used in a Zn clinical trial in Tanzania^(9)^, where caregivers were instructed how to open the blister pack and the capsule, dilute the Zn powder in small cup with 5 ml sterile water and feed the supplement to the child.

We assumed the supplementation intervention would be delivered through child clinics conducted routinely at heath facilities, with Zn supplementation provided together with other routine clinical services (growth monitoring, vaccination, nutrition counselling and general health education). Tanzanian children are recommended to attend clinic monthly for growth monitoring up to 5 years of age^(17)^. In practice, attendance is high until 18 months of age (1·5 years) a time when most of the vaccination schedule has been completed^(2)^. The supplement would be distributed to caretakers at two contacts. The first distribution would occur at the 6 weeks visit and dispense 8 months’ worth of Zn supplementation. The second distribution would occur at the 9 months visit, dispensing a subsequent 9 months’ worth of Zn supplementation. Instruction on dosage, timing and how to prepare the supplement would be provided each time the supplement is provided to the caregiver.

### Effectiveness of Zn in preventing diarrhoea

The effectiveness of Zn in preventing diarrhoea was established by a large clinical trial in Tanzania^(9)^. Information on rate ratios for diarrhoeal incidence was obtained from the trial (Table 1) and we estimated the incremental difference in diarrhoea incidence over a 17-month analytic period (up to 18 months of age). The number of diarrhoea episodes averted was estimated as the difference in diarrhoea episodes with Zn supplementation compared with diarrhoea episodes without Zn supplementation. Total diarrhoea episodes without Zn were calculated as a product of the annual incidence rate for diarrhoea, the analytic period in years and cohort size. We calculated the total diarrhoea episodes with Zn supplementation by multiplying this total by the rate ratio associated with Zn supplementation. We assumed that adherence with the intervention in routine practice would be lower than observed in the trial setting^(18)^, and that reductions in compliance would reduce health benefits proportionally (e.g. 80 % compliance would reduce health benefits by 20 % compared with compliance in the trial).

**Table 1:** Parameter estimates used in the model

Category	Parameter description	Parameter value	95 % uncertainty interval	Distribution used for probabilistic sensitivity analysis	Source
Cohort	Cohort size		10 000	Fixed value	Assumed Zn distributed to 10 000 children > 6 weeks to 18 months old
Risk of diarrhoea and sequelae	Rate of diarrhoea episodes (per year)	1·530	1·243, 1·844	Gamma (99·4, 65·0)	^(47)^
	Average duration of diarrhoea, per episode (days)	6·4	4·3, 8·4	Gamma (37·3, 5·8)	^(48)^
	No. of outpatient visits per episode, diarrhoea	0·72	0·30, 0·78	Gamma (34·4, 47·8)	^(49)^
	Probability of hospitalisation with diarrhoea, per episode	0·064	0·027, 0·069	Beta (32·2, 472·3)	^(9,49)^
	Average number of bed days, per hospitalisation	4·0	2·0, 6·0	Gamma (15·2, 3·8)	^(50,51)^
	Case fatality for diarrhoea	0·000232	0·000142, 0·000379	Beta (14·5, 62 686)	Ratio of diarrhoeal mortality to diarrhoeal incidence in individuals under 5 years of age^(47)^
Intervention parameters	Risk ratio of diarrhoea with Zn supplementation	0·88	0·81, 0·96	Gamma (528·7, 600·8)	^(9)^
	Compliance with Zn supplementation (%)	81	70, 90	Beta (46·4, 10·9)	^(45,46)^
	Number of units of Zn dispensed		540	Fixed value	Total months multiplied by 30 d per month
Unit costs*	Unit cost of an outpatient visit (service provision, US$)	1·05	0·22, 3·30	Gamma (1·6, 1·5)	^(19)^
	Unit cost of an outpatient visit (medication and diagnostics, US$)	1·63	1·22, 2·04	Gamma (60·5, 37·1)	^(20)^
	Unit cost of an outpatient visit (caregiver lost productivity, US$)	1·47	0·73, 2·93	Gamma (6·7, 4·5)	^(20)^
	Unit cost of an outpatient visit (caregiver out-of-pocket costs, US$)	0·88	0·00, 1·61	Gamma (4·4, 5·0)	^(20)^
	Unit cost of a hospital bed day (service provision, US$)	4·79	1·93, 10·46	Gamma (4·7, 1·0)	^(19)^
	Unit cost of hospitalisation (medication and diagnostics, US$)	4·86	3·65, 6·08	Gamma (61·3, 12·6)	^(20)^
	Unit cost of hospitalisation (caregiver lost productivity, US$)	6·45	2·49, 10·47	Gamma (9·9, 1·5)	^(20)^
	Unit cost of hospitalisation (caregiver out-of-pocket costs, US$)	12·95	8·93, 16·91	Gamma (40·3, 3·1)	^(20)^
	Unit cost of Zn supplementation (30 d supply, US$)	0·61	0·31, 0·92	Gamma (15·2 24·9)	^(13,15,51)^
Other inputs	Years of life lost (YLL) per death in target population		85·21	Fixed value	^(26)^
	Disability weight for diarrhoea, mild	0·074	0·049, 0·104	Gamma (27·6, 373·5)	^(27)^
	Disability weight for diarrhoea, severe	0·247	0·164, 0·348	Gamma (27·5, 111·4)	^(27)^
	Discount rate		3 %	Fixed value	^(28)^

* All costs reported in 2019 US dollars·

### Cost of Zn supplementation and care for diarrhoeal illness

We estimated the average cost of Zn supplementation based on the raw materials cost (Zn and packaging). These costs were calculated as the daily cost of Zn multiplied by the number of days supplemented and number of children supplemented. As Zn would be delivered during routine clinic visits, we assumed there would be no additional visits required to deliver the intervention. The costs associated with episodes of diarrhoeal illness were calculated to determine cost savings that may be realised from Zn supplementation. For each strategy (supplementation *v*. no supplementation) we estimated the total cost of outpatient care as the product of the total number of diarrhoeal episodes, the number of clinic visits per episode and the cost per clinic visit. We also estimated the costs of hospitalisation associated with diarrhoeal care, calculated as the product of the total number of diarrhoeal episodes, the probability of hospitalisations per episode, the average duration of hospitalisation and the cost per-bed day for hospitalisation. For both outpatient and hospital-based care, cost inputs included provider costs (medications and service provision) and costs borne by caregivers (out-of-pocket spending and productivity losses). Sources of cost data included secondary cost data from WHO-CHOICE^(19)^ and similar studies^(10,13,15,19–21)^. Input values and sources are shown in Table 1.

### Cost adjustment

We adjusted costs to be representative of 2019 Tanzanian price levels^(22)^. For cost inputs obtained from other settings^(20)^, we adjusted for price differences between settings using health sector price indices reported by the World Bank’s International Comparison Program^(23)^. To adjust for inflation, we first converted cost inputs into Tanzanian Shillings using exchange rates reported by the Bank of Tanzania for the years in which cost data were collected and then inflated costs to 2019 levels using the GDP deflator^(24)^. We converted the resulting cost estimates to US dollars using the 2019 exchange rate^(25)^. All results are reported in 2019 US dollars.

### Disability-adjusted life years averted by Zn supplementation

Disability-adjusted life years (DALY) averted were calculated to quantify health outcomes. DALY estimation considered years of life lost due to premature mortality and years lived with disability due to non-fatal health losses resulting from diarrhoea. years of life lost were calculated as the product of the estimated number of diarrhoeal deaths (total diarrhoeal episodes multiplied by case fatality) and standard life expectancy at the age of diarrhoeal death^(26)^. To calculate years lived with disability, we multiplied the number of person-years spent with diarrhoea (total diarrhoeal episodes multiplied by average disease duration) by the disability weight for mild diarrhoea^(27)^. To this we added the number of person-years spent hospitalised due to diarrhoea (number of diarrhoea hospitalisations multiplied by average duration of hospitalisation) multiplied by the disability weight for severe diarrhoea. Parameter inputs for calculating DALY were obtained from the Zn trial, published literature and standard values used in the Global Burden of Disease study (Table 1). DALY were discounted using a 3 % discount rate^(28)^.

### Cost-effectiveness of Zn supplementation

We estimated the incremental cost-effectiveness ratio (ICER) comparing Zn supplementation to the standard-of-care. The primary cost-effectiveness outcome was the incremental cost per DALY averted, with costs assessed from a societal perspective and a discount rate of 3 % applied to future outcomes^(28)^. We also calculated the incremental cost per DALY averted from a health system perspective (omitting patient-incurred costs) and present both outcomes with and without discounting. We also report the cost per diarrhoea episode averted, from societal and health system perspectives, without discounting.

### Uncertainty and sensitivity analysis

We conducted several sensitivity analyses to describe the robustness of model results to changes in model parameters^(29)^. Firstly, we conducted deterministic one-way sensitivity analyses for each model parameter. To do so, we varied each parameter between its upper and lower bounds (Table 1), while holding other parameters at their mean value, and recorded the resulting change in the primary ICER outcome. Secondly, we conducted a probabilistic sensitivity analysis to describe the combined impact of uncertainty in all model parameters. We specified closed-form prior distributions for each model parameter (beta distributions for probabilities and other parameters defined between zero and one, and gamma distributions for risk ratios and other non-negative parameters), with the mean and dispersion of these distributions chosen to reproduce the mean and interval widths for each parameter (Table 1). Using these distributions, we drew a Latin hypercube sample of 100 000 parameter sets and performed a second-order Monte Carlo simulation. With the results produced, we calculated equal-tailed 95 % uncertainty intervals (UI) for each major outcome^(29)^. We also used these results to calculate cost-effectiveness acceptability curves^(30)^, describing the probability that Zn supplementation is cost-effective for different threshold values for the cost per DALY averted.

To draw conclusions about cost-effectiveness, we compared ICER and cost-effectiveness acceptability curves to conventional cost-effectiveness thresholds, with a cost-effectiveness ratio below the threshold indicating that is cost-effective. The thresholds included 1 times and 3 times per capita GDP, which have been proposed by the Commission on Macroeconomics and Health^(31)^ and subsequently adopted by WHO-CHOICE for resource allocation decisions^(32)^, as well as more recently published thresholds that attempt to quantify the opportunity costs of reallocating spending within the health budget^(33,34)^. These recent thresholds produce more stringent criteria for identifying an intervention as cost-effective.

In addition, we examined how results changed when we used estimates from a recent meta-analysis to parameterise the effect of Zn supplementation^(35)^, instead of the estimates reported by McDonald *et al*. In this meta-analysis, prophylactic Zn was found to reduce diarrhoea incidence with a rate ratio of 0·89 (95 % CI 0·82, 0·97) and reduce the average duration of diarrhoeal episodes by 0·5 d.

## Results

### Cost of Zn supplementation

Zn supplementation was estimated to cost $109 800 (95 % UI 61 716, 171 507) for the study cohort of 10 000, equivalent to $11 per child. The provider cost of diarrhoea treatment (outpatient and hospitalisation) was estimated to be $78 347 (95 % UI 42 694, 136 095) without Zn supplementation and $70 734 (95 % UI 38 286, 123 212) with Zn supplementation, equivalent to cost savings of $7613 (95 % UI 2295, 15 878) with Zn supplementation, or $0·76 per child. The cost incurred by patients in their families for diarrhoea treatment (outpatient and hospitalisation), which includes out-of-pocket spending and lost productivity, was estimated to be $66 312 (95 % UI 39 875, 103 535) without Zn supplementation and $59 868 (95 % UI 35 728, 93 837) with Zn supplementation, equivalent to cost savings of $6444 (95 % UI 2045, 12 529) with Zn supplementation, or $0·64 per child. Combining patient and health services costs, total cost savings from averted diarrhoea care were $12 887 (95 % UI 4089, 25 058). Additional cost outcomes are described in Table 2.

**Table 2:** Outcomes and costs for standard-of-care and Zn supplementation strategies

Outcome	Standard-of-care		Zn supplementation		Incremental difference*	
	OR	95 % CI	OR	95 % CI	OR	95 % CI
Number of diarrhoeal episodes	22 636	18 406, 27 297	20 435	16 426, 24 911	–2200	–3737, –776
Number of outpatient visits for diarrhoea	16 297	10 704, 23 319	14 713	9598, 21 178	–1584	–2927, –522
Number of hospitalisations for diarrhoea	1444	946, 2065	1304	848, 1878	–140	–259, –47
Number of hospital bed days for diarrhoea	5775	2808, 10 152	5214	2526, 9227	–561	–1189, –160
Number of deaths from diarrhoea	5·251	2·788, 8·569	4·741	2·509, 7·769	–0·510	–1·029, –0·152
Number of days with diarrhoea	144 866	96 555, 205 331	130 786	86 570, 186 518	–14 080	–25 839, –4692
YLD due to diarrhoea	33·3	19·8, 52·2	30·0	17·8, 47·3	–3·2	–6·3, –1·0
YLL due to diarrhoea	447·5	237·6, 730·2	404·0	213·7, 662·0	–43·5	–87·6, –12·9
YLL due to diarrhoea, discounted at 3 %	165·8	88·0, 270·5	149·7	79·2, 245·2	–16·1	–32·5, –4·8
Health system costs of outpatient diarrhoea care	43 671	21 569, 85 433	39 428	19 401, 77 295	–4243	–9627, –1203
Patient costs of outpatient diarrhoea care	38 295	16 831, 71 040	34 574	15 155, 64 368	–3721	–8224, –991
Health system costs of hospitalisation for diarrhoea	34 676	12 857, 76 823	31 306	11 574, 69 398	–3370	–8504, –795
Patient costs of hospitalisation for diarrhoea	28 018	16 459, 43 843	25 295	14 774, 39 822	–2723	–5312, –857
Cost of Zn supplement	0	0, 0	109 800	61 716, 171 507	109 800	61 716, 171 507

* Calculated as Zn supplementation minus base case. All costs reported in 2019 US dollars.

### Effectiveness of Zn supplementation

Zn supplementation was estimated to avert 2200 (95 % UI 776, 3737) episodes of diarrhoea, 1584 (95 % UI 522, 2927) outpatient visits, 561 (95 % UI 160, 1189) hospital bed days, 0·51 (95 % UI 0·15, 1·03) deaths and 14 080 (95 % UI:4692 ,25 839) sick days. Taken together, this resulted in 3·2 (95 % UI 1·0, 6·3) fewer years lived with disability and 43·5 (95 % UI 12·9, 87·6) fewer years of life lost. Detailed health outcomes for each strategy are reported in Table 2. In aggregate, we estimated 46·7 (95 % UI 14·3, 92·6) fewer DALY for the Zn supplementation strategy compared with no supplementation (equivalent to 19·3 (95 % UI 6·1, 37·5) discounted DALY) (Table 3).

**Table 3:** Cost-effectiveness results

Outcome	Base case		Zn supplementation		Incremental difference*	
	OR	95 % CI	OR	95 % CI	OR	95 % CI
DALY due to diarrhoea	480·7	268·4, 767·8	434·0	241·8, 694·2	–46·7	–92·6, –14·3
DALY due to diarrhoea, discounted at 3 %	199·0	117·9, 307·9	179·7	106·1, 279·0	–19·3	–37·5, –6·1
Total costs (health system perspective)	78 347	42 694, 136 095	180 534	118 790, 258 104	102 187	53 410, 164 354
Total costs (societal perspective)	144 659	91 185, 220 646	240 403	168 001, 329 811	95 744	46 209, 158 172
ICER (health system perspective, undiscounted)					2187	806, 7808
ICER (societal perspective, undiscounted)					2049	686, 7624
ICER (health system perspective, discounted)					5283	1976, 18 335
ICER (societal perspective, discounted)					4950	1678, 17 933

* Calculated as Zn supplementation minus base case. All costs reported in 2019 US dollars.

### Incremental cost-effectiveness ratios for Zn supplementation

**Fig. 1:**
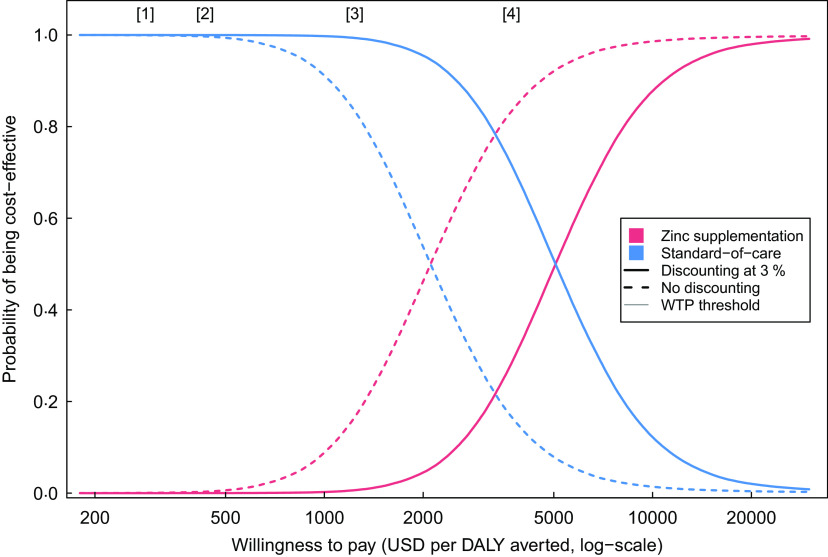
Cost-effectiveness acceptability curve for Zn supplementation *v.* no supplementation*. * Results calculated from the societal perspective, with and without future outcomes discounted at 3 % (the discounted results represent the primary cost-effectiveness outcome). Vertical lines indicate published cost-effectiveness thresholds: [1] Opportunity-cost threshold proposed by Woods *et al*.^(33)^ based on midpoint of range of published values and equivalent to 23 % of per capita GDP; [2] opportunity-cost threshold proposed by Ochalek *et al*.^(34)^, based on ‘method 4’ in the published article and equivalent to 35 % of per capita GDP; [3] historical threshold of 1 times per capita GDP for identifying an intervention as ‘very cost-effective’, as adopted by the WHO-CHOICE project^(32)^; [4] historical threshold of 3 times per capita GDP for identifying an intervention as ‘cost-effective’, as adopted by the WHO-CHOICE project^(32)^

For the primary cost-effectiveness outcome (societal perspective, 3 % discount rate applied to future outcomes) the ICER of Zn supplementation was $4950 (95 % UI 1678, 17 933) per DALY averted, as compared with no supplementation. Without discounting the ICER was substantially lower, $2049 (95 % UI 686, 7624) per DALY averted, reflecting the time lag between intervention spending and the life years gained through reduced diarrhoeal mortality. ICER estimated from a health system perspective were similar to the results from the societal perspective (Table 3). The incremental cost per diarrhoeal episode averted was $57 (95 % UI 19, 145) from a societal perspective and $54 (95 % UI 16, 142) from a health system perspective. Figure 1 shows the probability that Zn supplementation is cost-effective compared with no supplementation, for different values of the cost-effectiveness threshold, with and without discounting, and compared with different criteria for identifying an intervention as cost-effective.

### Sensitivity analyses

**Fig. 2:**
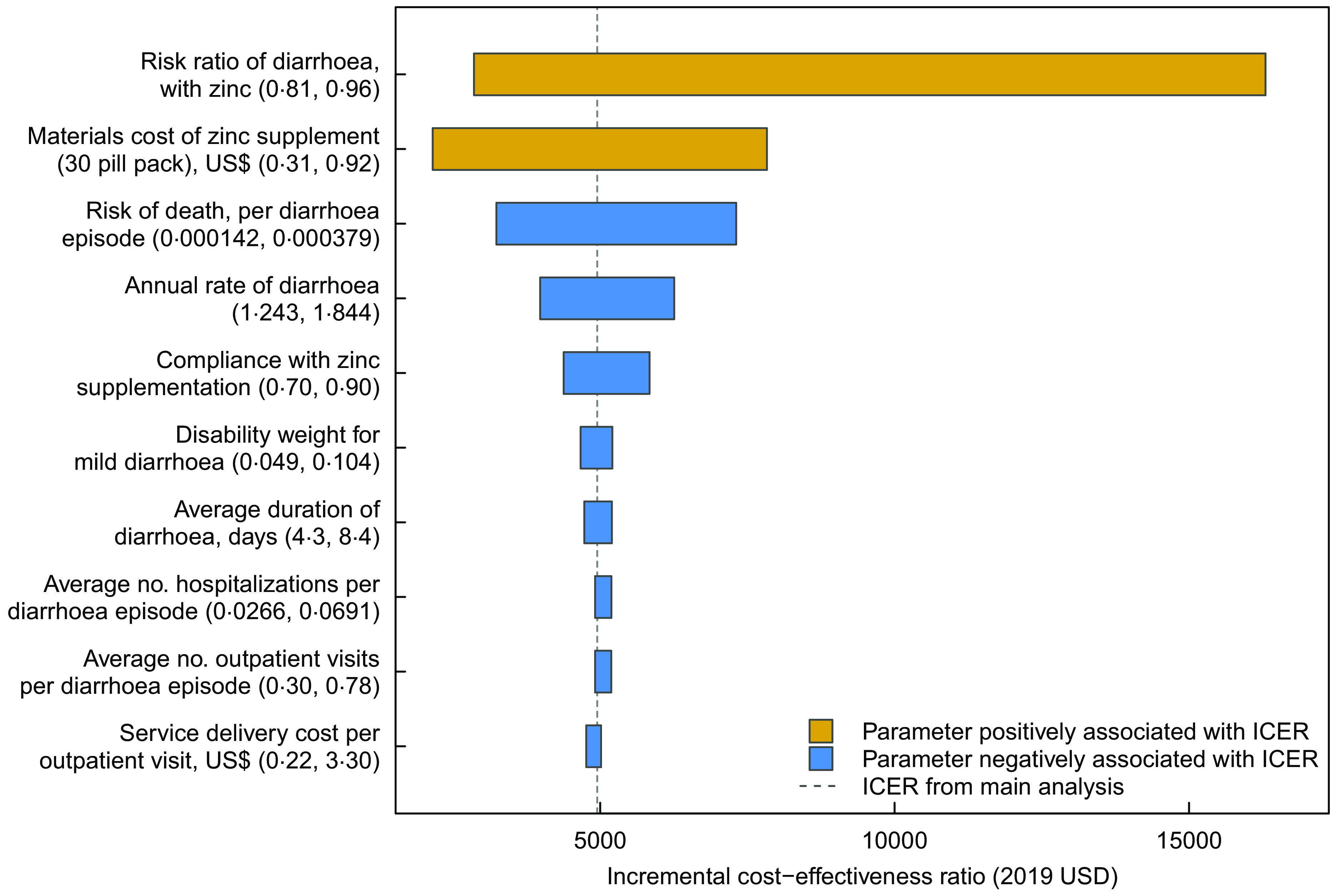
Results of one-way sensitivity analyses*. * Incremental cost-effectiveness ratio (societal perspective, discounted at 3 %)

We conducted deterministic one-way sensitivity analyses for each model parameter. Figure 2 summarises these results for the ten most influential parameters. Of these parameters, uncertainty in the risk ratio of diarrhoea with Zn supplementation had the greatest impact on the cost-effectiveness results, with the cost per DALY averted rising from $2857 to $16 289 as this parameter was varied from its lowest to highest value. The unit cost of Zn supplementation was also influential, with the cost per DALY averted rising from $2157 to $7832 as this parameter was varied from its lowest to highest value.

When we re-ran the analysis using input data from^(35)^, we found very similar results compared with the main analysis, with a cost per DALY averted of $4890 (95 % UI 1715, 16 328) for the primary cost-effectiveness outcome, 1 % lower than the ICER estimated in the main analysis.

## Discussion

In this study, prophylactic Zn supplementation for infants and children in Tanzania was shown to reduce the health losses, healthcare utilisation and productivity losses associated with childhood diarrhoea. This included averting episodes of diarrhoea, reducing outpatient visits and inpatient days for diarrhoea, reducing sick days and reducing diarrhoeal deaths in the study population. These results are consistent with other studies that have estimated the benefits of Zn supplementation for therapy as well as prevention. For example, recent clinical trials conducted in Tanzania have shown that daily Zn supplementation for infants can reduce the burden of diarrhoea^(9,36)^. Other similar studies have reported reduced episodes of diarrhoea^(7,37,38)^, reduced duration of diarrhoea^(38–40)^, reduced severity of diarrhoea^(38)^, reduced diarrhoea mortality^(5,7)^ and reduced mean cost of diarrhoea treatment^(39)^.

However, our results also show relatively high costs for implementing prophylactic Zn supplementation. The mean cost of supplementation was $11 per child. Other studies have reported high cost of Zn supplementation as well^(41)^. Driven by these high implementation costs, our results demonstrate that prophylactic Zn supplementation may not be cost-effective for the prevention of childhood diarrhoea in settings like Tanzania. While there is considerable uncertainty about the appropriate cost-effectiveness threshold to apply in low- and middle-income countries, most conventional estimates suggest that interventions should have a cost per DALY averted that is substantially below a country’s per capita GDP to be considered cost-effective^(33,34)^. The primary cost-effective ratio estimated in this study – $4950 per DALY averted – is well above the per capita GDP in Tanzania (US$1122 in 2019^(42)^), suggesting that the health and economic benefits produced by reduced diarrhoea incidence do not justify the costs required to implement the intervention. Even with more relaxed thresholds of 1 times and 3 times per capita GDP – now thought to be a poor representation of the opportunity cost of healthcare spending in settings like Tanzania^(43)^ – supplementation does not appear cost-effective.

In sensitivity analysis, we found that this cost-effectiveness ratio was most sensitive to the unit costs of supplementation, as well as the risk ratio for the reduction in diarrhoeal risk with supplementation. As a consequence, this intervention could become cost-effective if there were price reductions for the Zn tablets, or if accumulating evidence suggested greater risk reductions for supplementation. There are good reasons to believe that lower intervention costs are possible. Firstly, the cost of Zn supplementation could go down in light of the findings from a recent large clinical trial carried out among children in Tanzania and India, which found that lower doses of Zn (5 and 10mg) were non-inferior with respect to duration of diarrhoea and number of stools during an episode, compared with the WHO recommended 20 mg dose^(36)^.

Despite the lack of cost-effectiveness of the Zn supplementation scenario presented in our modelling findings, many studies have suggested Zn supplementation may be of benefit in high diarrhoea burden areas where childhood Zn deficiency and malnutrition are prevalent^(5,40)^. It is possible that an intervention with a more favourable cost-effectiveness ratio could be achieved by targeting prophylactic Zn supplementation to lower income households with a high burden of childhood malnutrition and diarrhoea.

A major strength of this study was the availability of local data required for the analysis. This allowed us to parameterise the study model with evidence collected in the setting of interest, including the clinical trial that estimated the risk ratio of diarrhoeal incidence attributable to Zn supplementation^(9)^. Another strength of the study is that it responds to a clear gap in the evidence base for Zn supplementation, as there are very few studies on the cost-effectiveness of prophylactic Zn supplementation^(13)^. Moreover, cost-effectiveness studies on therapeutic Zn have reported inconsistent results, with some reporting cost-effectiveness of Zn in treating childhood diarrhoea^(13,39)^, while other report no cost-effectiveness^(41)^.

This study also has several limitations. First, the major outcomes (changes in DALY and diarrhoeal mortality) were not estimated empirically but instead relied on decision-analytic modelling to extrapolate outcomes, based on expected differences in diarrhoeal incidence. While modelling plays an important role in generalising the results of empirical trials, the construction of models requires additional assumptions. In this study, major assumptions were the base rate of diarrhoeal illness, and the case fatality associated with diarrhoeal illness. For this reason, further empirical assessment of the cost and health impacts of prophylactic Zn supplementation would add robustness to the findings of this study. Second, we did not model the potential health benefits of prophylactic Zn on pneumonia incidence. The evidence base supporting preventive effects for pneumonia is relatively weak, with^(35)^ reporting a risk ratio of 0·87 (95 % CI 0·81, 0·94) for pneumonia incidence, with a low GRADE assessment. However, if prophylactic Zn does prevent pneumonia, this could substantially improve cost-effectiveness, as pneumonia causes many more under-5 deaths and DALY in Tanzania than diarrhoea^(44)^. For this reason, our cost-effectiveness results should be revisited if evidence confirms a protective effect for Zn supplementation against pneumonia. Third, although the Tanzanian Zn supplementation trial reported a statistically significant reduction in diarrhoea incidence, there were no statistically significant differences observed for outpatient visits, hospitalisations and mortality^(9)^. In contrast, our modelling allowed for reductions in these outcomes proportional to the effect on diarrhoea incidence. As these other outcomes have a lower base rate in the study cohort, it is not surprising that statistically non-significant differences were reported for these outcomes, due to the relatively modest rate ratio for diarrhoea (0·78) reported by the study. However, if Zn supplementation truly has no effect on these outcomes, it would make Zn supplementation substantially less cost-effective. Fourth, it is possible that the analysis did not sufficiently adjust for the lower level of compliance in routine settings compared with the trial. In the analysis, we assumed compliance would be 81 % (70, 90)^(45,46)^. If compliance were to be substantially lower than this, the cost-effectiveness of Zn supplementation would be worse, as intervention costs would be unchanged but health benefits proportionally lower.

### Conclusion

Prophylactic Zn has positive health and economic benefits; however, it was not found to be cost-effective for prevention of childhood diarrhoea in the scenarios examined in this study. Additional research is needed to identify intervention approaches that can achieve the health benefits of prophylactic Zn supplementation at lower implementation costs.
